# Redirecting mouse T hybridoma against human breast and ovarian carcinomas: *in vivo* activity against HER-2/neu expressing cancer cells

**DOI:** 10.1038/sj.bjc.6600888

**Published:** 2003-04-15

**Authors:** A D Gritzapis, A Mamalaki, A Kretsovali, J Papamatheakis, M Belimezi, S A Perez, C N Baxevanis, M Papamichail

**Affiliations:** 1 Saint Savas Cancer Hospital, Cancer Immunology and Immunotherapy Center, 171 Alexandras Ave, 11522 Athens, Greece; 2 Department of Biochemistry, Hellenic Pasteur Institute, Athens, Greece; 3 Foundation for Research and Technology, Institute of Molecular Biology and Biotechnology, Heraklion, Crete

**Keywords:** chimeric receptor, cancer immunotherapy, HER-2/neu

## Abstract

Chimeric receptors comprising of the T-cell receptor-*ζ* cytoplasmic signalling chain fused to an extracellular ligand-binding domain of a single-chain antibody (scFv) have served as effective tools for redirecting cytotoxic T lymphocytes (CTL) against tumour cells. In this report, we constructed a chimeric scFv/*ζ* gene composed of the variable regions of an HER-2/neu-specific monoclonal antibody (MAb) joined to the TCR-*ζ* chain. The scFv(anti-HER-2/neu)/*ζ* chimeric gene was successfully expressed as a functional surface receptor in the MD.45 CTL hybridoma (MD.45-HER/*ζ*). More importantly, the scFv(anti-HER-2/neu)/*ζ* receptor was functionally active, since it triggered cytokine secretion by the MD.45-HER/*ζ* cells upon recognition of HER-2/neu-positive (+) tumour cell lines, or primary tumour cells from patients with HER-2/neu^+^ cancers. The MD.45-HER/*ζ*-transduced cells also lysed HER-2/neu^+^ target cells *in vitro* with high specificity. We tested the antitumour efficacy of scFv(anti-HER-2/neu)/*ζ* expressing MD.45 cells in severe combined immunodeficiency disease mice/human and murine tumour models. The adoptively transferred MD.45-HER/*ζ* cells both slowed significantly the growth of human FM3 melanoma or murine ALC leukaemic cells both transfected to express HER-2/neu. Our data demonstrate the feasibility of redirecting MD.45 CTL with the scFv(anti-HER-2/neu)/*ζ* chimeric receptor to respond specifically against HER-2/neu expressing tumour cells *in vitro* and *in vivo*. Moreover, they make it likely that T cells transduced with the same chimeric gene might be utilised in the treatment of patients with HER-2/neu^+^ tumours.

A general limitation in the use of cellular adoptive immunotherapy is the difficulty in obtaining high numbers of lymphocytes specifically responding to autologous tumour cells. Nevertheless, in some cases, monoclonal antibodies (MAb) against tumour-associated antigens have been successfully generated (Abken *et al*, 1998). One approach, merging T-cell functions with antibody specificity, was to create chimeric receptors composed of a single-chain variable domains of MAb (scFv) fused to signalling *ζ*-chain of the T-cell receptor (TCR) (Moritz *et al*, 1994; Altenschmidt *et al*, 1997; Brocker and Karjalainen, 1997; Maher *et al*, 2002; Niederman *et al*, 2002) or the *γ*-chain of the low-affinity receptors for IgG (Fc*γ*RIII) (Eshhar *et al*, 1993,^2001^; Hwu *et al*, 1995; Roberts *et al*, 1998) or IgE (Fc*ɛ*RI) (Weijtens *et al*, 1996; Haynes *et al*, 2001). Introduction of such chimeric genes into T cells enables them to respond in an MHC-independent fashion to an antigen-specific trigger via these receptors by cytokine production (Altenschmidt *et al*, 1997; Eshhar *et al*, 2001; Maher *et al*, 2002) and tumour cell lysis (Eshhar *et al*, 1993; Moritz *et al*, 1994; Hwu *et al*, 1995; Weijtens *et al*, 1996).

The HER-2/neu oncogene (also known as ErbB-2) encodes a 185 kDa transmembrane protein-receptor with tyrosine kinase activity and extensive homology to the epidermal growth factor receptor (Hung and Lau, 1999). HER-2/neu is ubiquitously expressed in many epithelial tumours and known to be overexpressed in approximately 30% of all ovarian and breast cancers (Slamon *et al*, 1989,^2001^), 35–45% of all pancreatic carcinomas (Yamanaka *et al*, 1993) and up to 90% of colorectal carcinomas (Maxwell-Armstrong *et al*, 1998) and this overexpression was shown to correlate with aggressiveness of malignancy and poor prognosis (Slamon *et al*, 1989; Pauletti *et al*, 1996). The HER-2/neu protein appears to be immunogenic because T-lymphocyte responses to both MHC class I- and class II-restricted epitopes have been observed (Yoshino *et al*, 1994; Fisk *et al*, 1995; Peiper *et al*, 1997; Brossart *et al*, 1998; Rongcun *et al*, 1999; Sotiriadou *et al*, 2001; Baxevanis *et al*, 2002; Perez *et al*, 2002). However, the use of such peptide epitopes for vaccination studies is limited to only those patients who express the appropriate MHC class I and class II alleles. The generation of T lymphocytes with a grafted MHC-independent recognition specificity for HER-2/neu expressing tumour cells could probably circumvent this problem. To this end the *ζ*-chain of the TCR was linked with a scFv derived from a MAb directed against the human ErbB-2 receptor (Moritz *et al*, 1994; Altenschmidt *et al*, 1997). The scFv(ErbB-2)/*ζ* fusion gene was stably expressed in murine T lymphocytes that subsequently could recognise and lyse either mouse cell lines transfected to express the human ErbB-2 receptor or the human breast cancer MDA-MB453 cell line constitutively expressing ErbB-2 (Moritz *et al*, 1994; Altenschmidt *et al*, 1997). The same chimeric construct was recently used for redirecting a human NK cell line against HER-2/neu^+^ tumours (Uherek *et al*, 2002).

In the present study, we constructed a novel HER-2/neu recognising chimeric receptor by fusing a scFv derived from an anti-human HER-2/neu MAb produced by the HB8696 hybridoma with the *ζ*-chain of the TCR. Such chimeric genes were stably transduced in the murine MD.45 cytotoxic T lymphocytes (CTL) hybridoma, which could specifically recognise and lyse *in vitro* HER-2/neu expressing human tumour cell lines as well as metastatic tumour cells from different types of cancer. The transduced MD.45 CTL were also active *in vivo* in that they slowed tumour growth in severe combined immuno deficiency disease (SCID) mouse/human tumour models. Our data support the use of our scFv(anti-HER-2/neu)/*ζ* chimeric receptor in protocols related to combined cellular and gene therapy of cancer.

## MATERIALS AND METHODS

### Patients

Patients with metastatic breast and ovarian adenocarcinomas (stages III and IV) whose tumours expressed HER-2/neu (four of 17 examined; breast Ca, *n*=2; ovarian Ca, *n*=2) were included in this study. Ascites from these patients collected during routine aspirations were provided by the Oncology Department of Hippocration State Hospital and the Pathological Clinics of Saint Savas Cancer Hospital under the Institutional Review Boards of both Institutions.

### Cell lines

The human breast cancer SKBR3 and ovarian cancer SKOV3 cell lines both expressing HER-2/neu (Brossart *et al*, 1998; Sotiriadou *et al*, 2001) as well as the Raji (Burkitt's lymphoma) and K562 (erythroleukemia) cell lines were purchased from the American Type Culture Collection (ATCC) (Manassas, VA, USA). The mouse hybridoma cell lines Myc-9E10.2 secreting a c-myc-specific MAb (Evan *et al*, 1985) and HB8696 secreting the MAb 520C9 which recognises the human c-ErbB-2 oncoprotein (Ward *et al*, 1989; Shi *et al*, 1991) were also purchased from the ATCC. The MD.45 CTL hybridoma of BALB/c origin was kindly provided by Dr Z Eshhar (The Weizmann Institute of Science, Rehovot). The human FM3 melanoma cell line and the murine ALC lymphoma cell line were kindly provided by Dr J Zeuthen (Danish Cancer Society Research Center, Copenhagen) and by Dr R Kiessling (Microbiology and Tumour Biology Center, Karolinska Institute, Stockholm) respectively. The ALC cell line was grown *in vivo* as ascites by serial passages in C57BL/6 syngeneic mice. All other cell lines were grown in RPMI-1640 supplemented with 10% fetal bovine serum (Life Technologies, Gaithersburg, MD, USA), 2 mM L-Glutamine and 50 *μ*g/ml gentamycin (both from Sigma, St Louis, MO, USA) (complete medium).

### Isolation of tumour cells

This was performed as recently described (Baxevanis *et al*, 2000). Ascites were centrifuged at 400 g for 5 min to sediment cells, which were further placed on top of a 75% Ficoll–Hypaque gradient, overlaid on 100% Ficoll–Hypaque, and spun at 700 g for 25 min. Tumour cells were collected from the top of 75% Ficoll–Hypaque and cryopreserved in liquid nitrogen until ready for use. At that time, cells were carefully thawed, slowly diluted in RPMI-1640 (Life Technologies, Gaithersburg, MD, USA) and washed. Tumour cells were assayed only if their viability was over 80%. Phenotype analysis showed that all tumours were positive after staining with an anti-HER-2/neu MAb (clone Neu 24.7) and anti-mouse FITC (both, Becton Dickinson Mountain View, CA, USA) (range of HER-2/neu expression: 18–92%; see also ‘Results’).

### Construction of scFv(anti-HER-2/neu)

Total cellular RNAs were isolated from hybridoma cells HB8696 with the hot-acid phenol method (Brown and Kafatos, 1988; Mamalaki *et al*, 1993). Specific first-strand VH- and VL-cDNAs were synthesised by the primer extension method using M-MLV reverse transcriptase (Stratagene) and specific oligonucleotide primers according to EMBO Practical curse (1991). For the construction of scFv fragment of anti-HER-2/neu, we amplified specifically the VH- and VL-cDNAs that were then assembled as a scFv fragment as described (Mamalaki *et al*, 1993), by using *Taq* DNA polymerase (Minotec, Heraclion, Greece). The assembled scFv fragment was inserted into the phagemid pHEN1 (Hoogenboom *et al*, 1991) which was used to transform *E. coli* HB2151. The pHEN1 phagemid contains the c-myc tag peptide and the produced scFv is thus a tag antibody fragment. All DNA manipulations were performed according to previously described techniques (Kabat *et al*, 1991). DNA sequences were determined by the dideoxy-chain termination method (Sanger *et al*, 1977) using Sequenase version 2.0 (United States Biochemical Cleveland, CH).

### Expression and purification of scFv(anti-HER-2/neu) fragment

HB2151 cells transformed with the pHEN1-scFv(anti-HER-2/neu) phagemid were grown at 25°C in 2 × TY medium containing 100 *μ*g/ml ampicillin and 1% glucose. Soluble antibody fragments were isolated from the culture supernatants after induction with 1 mM IPTG for 16 h. Expression of scFv antibody fragments was assessed by size analysis and immunoreactivity. Supernatants (15 *μ*l) from the induced cultures were analysed by electrophoresis on 12% SDS–polyacrylamide gels (Laemmli, 1970) and the proteins electrotransferred onto Hybond-C membranes (The Radiochemical Centre Amersham, UK) for immunoblotting. Soluble scFv fragments were detected using a serum-free hybridoma culture supernatant containing MAb 9E10, directed against the c-myc tag, and HRP-conjugated goat anti-mouse IgG (1 : 500 dilution) (DAKO A/S Glostrup Denmark), as previously described (Hoogenboom *et al*, 1991). The tagged scFv was purified on a protein A–Sepharose column using the anti-c-myc tag MAb 9E10, as previously described (Tsantili *et al*, 1999) .

### Immunofluorescence analysis of scFv(anti-HER-2/neu) binding on tumour cell lines

This was performed in two steps: at first, tumour cells were harvested and washed in PBS. Then an amount of 100 *μ*l of 1 : 10 diluted scFv(anti-HER-2/neu) was added per one million cells and incubated on ice for 45 min. In the second step, tumour cells were extensively washed to remove excess of scFv(anti-HER-2/neu) and then incubated for 30 min on ice with anti-myc MAb 9E10 followed by an additional 30 min incubation with FITC-conjugated anti-mouse Fab (1 : 20 dilution) (DAKO). After two washes in PBS, cells were analysed on a FACScan (Becton Dickinson) flow cytometer and calculated using the LYSYS II software (Becton Dickinson).

### Construction of the scFv(anti-HER-2/neu)/ζ chimeric gene

To construct the scFv/*ζ* chimeric receptor we used the eukaryotic expression vector pCDNA3 (Invitrogen). The scFv was amplified by PCR from the pHEN1 vector and cloned between the *Hind* III and *Bam*HI sites. Next, a leader peptide sequence from Ig heavy chain was synthesised *in vitro* and linked to the scFv via the inherent Pst I site. The CD3 *ζ* chain containing 3 aa extracellular sequence in addition to the transmembrane and cytoplasmic portion was amplified from the human cDNA clone kindly provided by Dr GC Tsokos (Walter Reed Army Institute of Research, Washington DC, USA) and cloned between *Bam*HI and *Eco*RI in frame to the scFv. We also inserted a Flag epitope in the *Bam*H1 site in order to monitor the expression of the product. The chimeric scFv/*ζ* gene was subsequently digested by *Hind* III and *Eco*RI and cloned (as a blunt fragment) into the retroviral vector pLRNL containing the long terminal repeat from Moloney murine leukaemic virus and a neomycin resistance gene. The retroviral constructs were transfected into the amphotropic cell line Phoenix (Pear *et al*, 1993) using CaPO_4_. A stable amphotropic packaging cell line, Phoenix, was obtained after G418 selection. The amphotropic virus supernatants produced had a viral titre of approximately 5 × 10^3^ CFU ml^−1^, determined on the basis of neomycin resistance of infected NIH-3T3 cells. Such supernatants were collected and stored at −20°C.

#### Gene transduction and selection of gene-transduced cells

This was performed as described previously (Weijtens *et al*, 1996). In brief, 2 × 10^6^ cells of the MD.45 hybridoma cell line were cocultured for 72 h with a 70–80% confluent irradiated (2500 rad) monolayer of virus pLRNLscFv(anti-HER-2/neu)/*ζ* producing Phoenix cells in culture medium supplemented with 4 *μ*g ml^−1^ polybrene (Sigma Chemical Co., St Louis, MO, USA) and 500 IU ml^−1^ rIL-2. Subsequently, the gene-transduced MD.45 population (MD.45-HER/*ζ*) was selected for 4 days in culture medium containing 1 mg ml^−1^ G418, followed by an additional round of selection (5 days) in medium containing 0.4 mg ml^−1^ G418. After selection, the MD.45-HER/*ζ* cells were expanded in round-bottom 96-well microtitre plates (Costar, Cambridge, MA, USA) at 37°C in 5% CO_2_ in the presence of feeder cells, which consisted of irradiated (2500 rad) allogeneic EBV-transformed lymphoblastoid B cell lines. Cloning of the MD.45-HER/*ζ* cells was performed by limiting dilution at 3,1 and 0.3 cells well^−1^ in the presence of feeder cells in RPMI-1640 culture medium supplemented with 10% FCS, 300 IU ml^−1^ rIL-2, 4 mM L-glutamine, antibiotics and 1 *μ*g ml^−1^ PHA (Sigma). Mock-transduced MD.45 cells (MD.45-mock) were generated by transducing the parental MD.45 cell line with the vector (i.e., pLRNL) alone.

### Transfection of the FM3 and ALC cell lines

This was performed as described recently (Perez *et al*, 2002). In brief, FM3 melanoma cells were cotransfected with a pSV2-c-erbB2 construct (kindly provided by Dr. Mien-Chie Hung, Anderson Cancer Center, Houston, TX, USA) and a pSV2neo plasmid using DNA-CaPO_4_ coprecipitates. Selection with G418 was performed 2 days later, followed by cloning and subcloning of FM3 cells expressing HER-2/neu (referred to as FM3-HER). The same procedure was followed for transfecting the ALC cells with the pSV2-c-erbB2 construct (referred to as ALC-HER). Mock-transfectants of FM3 (FM3-mock) and ALC (ALC-mock) were generated by transfecting the parental cell lines with the plasmid (i.e., pSV2) alone.

### Expression of the chimeric scFv(anti-HER-2/neu)/ζ gene

Expression of the scFv(anti-HER-2/neu)/*ζ* on the surface of transduced MD.45 cells was evaluated by indirect immunofluorescence staining using the anti-Flag MAb (Sigma) and FITC-labelled anti-mouse Fab′ antibody. Data were analysed and calculated as above.

### Functional assays

These included cytokine production and cytotoxicity: transduced MD.45 cells (10^6^) were cultured with 10^6^ HER-2/neu^+^ or HER-2/neu^−^ cell lines in 24-well plates (Costar) for 24 h. Following incubation, supernatants were harvested and spun to remove cell debris. Levels of cytokine production (IL-2 and IFN-*γ*) were measured by ELISA (Diaclone Research, Besancon France) according to the manufacturer's instructions.

Cytotoxicity mediated by the MD.45-transduced effectors against tumour HER-2/neu^+^ or HER-2/neu^−^ targets was determined as described (Baxevanis *et al*, 2000). The percentage of cytotoxicity was calculated according to the following formula: % lysis=100 (test ^51^Cr release–spontaneous ^51^Cr release)/(maximum ^51^Cr release–spontaneous ^51^Cr release). Cytotoxicity values were considered to indicate significant recognition of a target, when the differences between mean values (from triplicate analyses) for percent lysis of the particular target (e.g., HER-2/neu^+^ tumour cell targets) and HER-2/neu^−^ targets were equal to or higher than 10% at an E : T ratio of 20 : 1 and statistically significant (*P*<0.05).

### Tumour therapy model

All the *in vivo* experimentations were carried out with approval form the ethical committee of St Savas Cancer Hospital and met all the standards required by the UKCCCR guidelines (Workman *et al*, 1998). The tumour therapy model was performed as follows: on day 1, CB-17 Prkdcscid /J (BALB/c scid) mice (Jackson Laboratory, Bar Harbor, Maine, Germany) 7–9 weeks of age received 1 × 10^6^ tumour cell s.c. injection. MD.45-HER/ζ or MD.45-mock CTL was injected i.p. (1 × 10^7^ cells injection^−1^) on three consecutive days (starting on day 1). Total observation was over 120 days. The observation was terminated with the euthanasia of mice when the tumour mass grew up to 1.5 cm in diameter.

## RESULTS

### Construction of scFv(anti-HER-2/neu)

**Figure 1:**
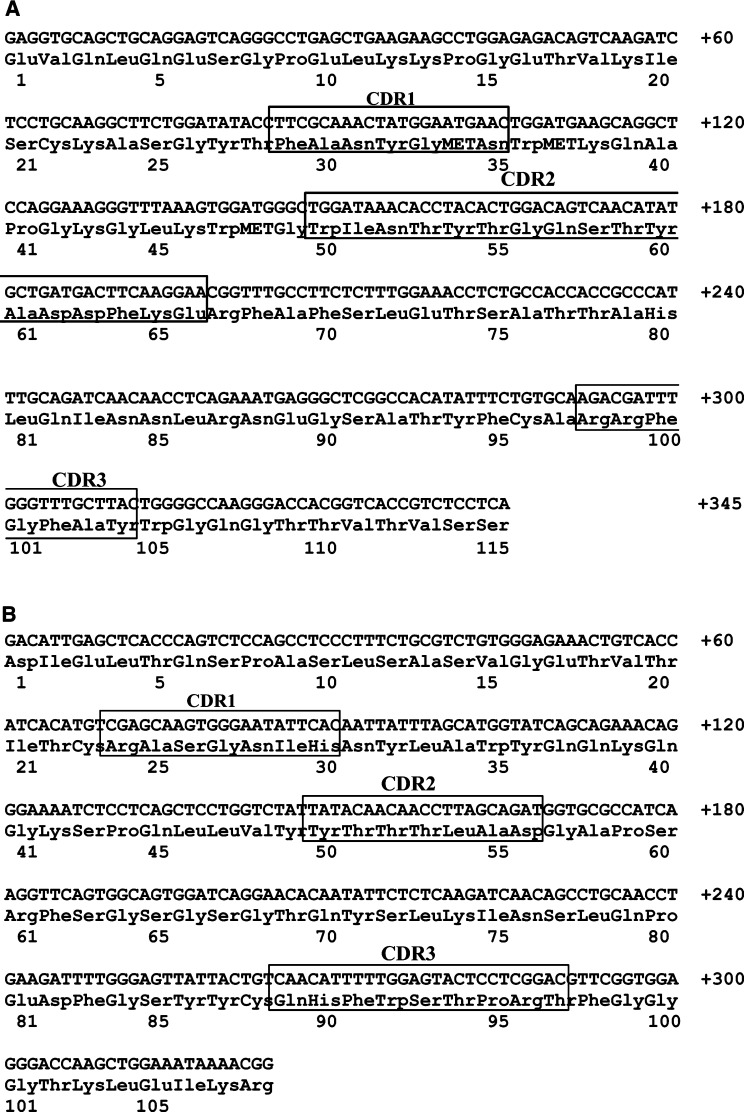
Nucleotide and deduced protein sequences of the heavy (**A**) and light (**B**) chain variable domain of MAb 520C9. The CDRs 1, 2 and 3 are indicated.

Specific VL- or VH-cDNAs of hybridoma HB8696 mRNA, enzymatically amplified, were assembled as scFv antibody fragment and cloned into pHENI vector (Hoogenboom *et al*, 1991). The primary structure of various clones was analysed by DNA sequencing. Figure 1A

shows the cDNA sequence and the deduced amino-acid sequences corresponding to the VH region of MAb 520C9. Sequence comparison showed that the VH region of MAb 520C9 is a member of the subgroup IIA according to Kabat *et al* (1991). The complementarity-determining region 2 (CDR2) is long, containing 17 amino-acid residues. Figure 1B shows the cDNA sequence and the deduced amino-acid sequences corresponding to the VL region of MAb 520C9. Sequence comparison showed that the VL region is a member of the Vk group (Kabat *et al*, 1991).

### Reactivity of the anti-HER-2/neu scFv with tumour cell lines and primary tumour cells

**Figure 2:**
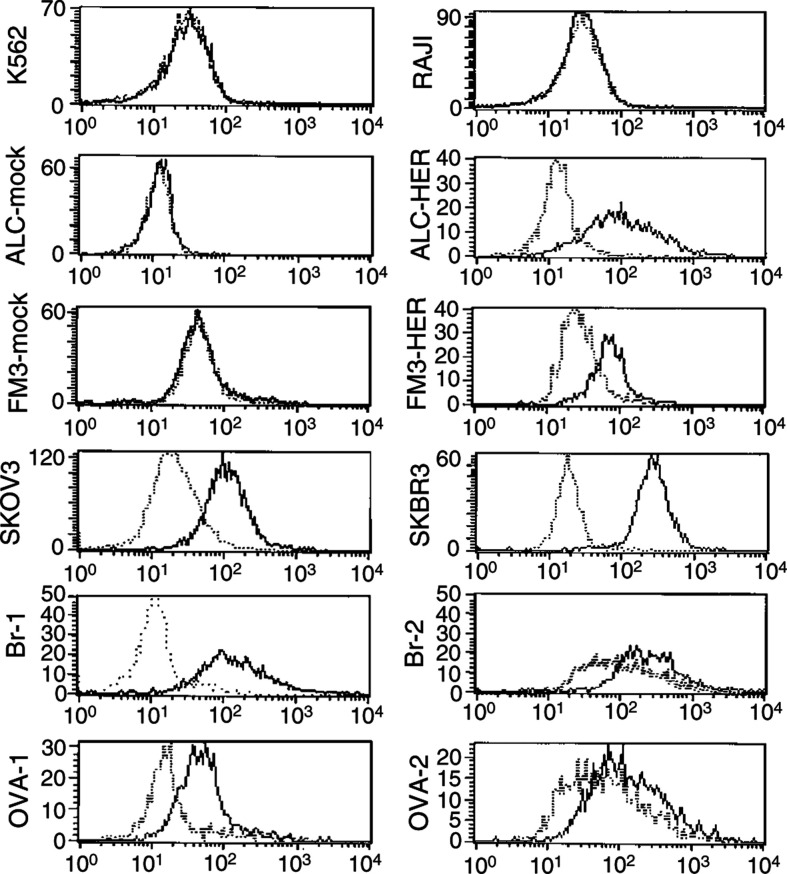
FACS analysis of immuofluorescence staining of HER-2/neu^+^ (ALC-HER, FM3-HER, SKBR3, SKOV3) and HER-2/neu^−^ (ALC-mock, FM3-mock, Raji, K562) tumour cell lines, treated with scFv(anti-HER-2/neu) and anti-myc MAb plus FITC-labelled anti-mouse Fab′ antibody. Ova-1, Ova-2, Br-1 Br-2 are HER-2/neu^+^ primary tumour cells from patients with metastatic ovarian and breast cancer. Solid lines: specific staining with scFv(anti-HER-2/neu) plus anti-myc and FITC-anti-mouse Fab′; dotted lines: staining with anti-myc and FITC-anti-mouse Fab′.

Binding of the scFv(anti-HER-2/neu) on tumour cells was determined by immunofluorescence and FACS analysis. Cell immunofluorescence was performed with ALC and FM3 tumour cell lines transfected to express HER-2/neu (ALC-HER and FM3-HER, respectively), their mock transfectants and with the SKBR3 and SKOV3 tumour cell lines constitutively expressing HER-2/neu. Primary HER-2/neu^+^ tumour cells from patients with metastatic breast (Br-1, Br-2) and ovarian (OVA-1, OVA-2) cancer were also analysed. The Raji and K562 cell lines, not expressing HER-2/neu, were included as negative controls. As shown in Figure 2

, the ALC-HER, FM3-HER, SKBR3 and SKOV3 tumour cell lines were positive in immunofluorescence staining with the scFv(anti-HER-2/neu) and anti-myc MAb plus FITC-anti-mouse Fab′ system (86%, 68%, 100% and 89%, respectively) and so did the primary tumour cells from metastatic breast (Br-1, 92%; Br-2, 27%) and ovarian (OVA-1, 81%; OVA-2, 18%) cancers. In contrast, ALC-mock and FM3-mock as well as Raji and K562 cell lines did not show positive staining demonstrating the ability of our scFv to specifically bind on HER-2/neu^+^ cells.

### Expression of the chimeric scFv(anti-HER-2/neu)/ζ gene

**Figure 3:**
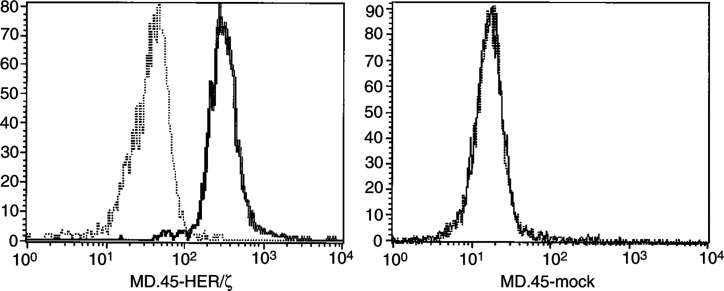
FACS analysis of immunofluorescence staining of MD.45 hybridoma transduced with pLRNLscFv(anti-HER-2/neu)/*ζ* (MD.45-HER/*ζ*) or with the vector alone (MD.45-mock). Expression of scFv(anti-HER-2/neu)/*ζ* was detected with an anti-Flag MAb plus FITC-labelled anti-mouse Fab′ (solid lines). Dotted lines: staining with FITC-labelled anti-mouse Fab′ alone.

Expression of the chimeric scFv(anti-HER-2/neu)/*ζ* receptor selective for HER-2/neu^+^ tumour cells was performed by constructing one continuous molecule comprising gene segments of the variable region of the murine anti-HER-2/neu MAb produced by the HB8696 hybridoma and the signal-transducing human TCR-*ζ* chain transmembrane and intracellular region. Introduction of the chimeric scFv(anti-HER-2/neu)/*ζ* gene into the MD.45-murine CTL hybridoma, resulted in the expression of the chimeric molecule on the cell surface of selected clones as revealed after staining with the anti-Flag-FITC MAb (Figure 3

).

### Cytokine secretion by the MD.45-HER/*ζ* cells upon recognition of HER-2/neu^+^ tumour cells or cell lines

The functionality of the chimeric receptor was examined by testing the ability of MD.45-HER/*ζ* cells to secrete IL-2 and IFN-*γ* upon interaction with HER-2/neu^+^ cells. As presented in Table 1

**Table 1:** Optimal IL-2 secretion of transduced MD.45-HER/*ζ* CTL upon target cell interaction

		**IL-2 production (pg ml^−1^)**		
**Targets**	**HER-2/neu expression^b^**	**MD.45-HER/*ζ***	**MD.45-HER/*ζ*+Neu 24.7**	**MD.45-mock**
ALC-mock^c^	—	22±5^d^	19±3 (13)^e^	<10
ALC-HER^c^	+	290±50	55±6 (81)	27±6
FM3-mock^e^	—	37±8	39±7 (−5)	32±10
FM3-HER^c^	+	368±32	67±12 (82)	39±6
SKBR3	+	1045±220	375±52 (64)	<10
SKOV3	+	960±180	295±27 (69)	23±5
OVA-1^f^	+	235±10	52±17 (78)	<10
OVA-2^f^	+	182±32	39±22 (78)	<10
Br-1^f^	+	650±75	105±23 (90)	42±8
Br-2^f^	+	268±29	106±17 (60)	<10
K562	—	<10	<10 (0)	<10
Raji	—	27±6	28±9 (−3)	<10

a MD.45 CTL transduced with scFv(anti-HER-2/neu)/*ζ* (MD.45-HER/*ζ*) was cultured at 1 × 10^6^ cells ml^−1^ with an equal number of tumour cell targets for 24 h in 24-well plates in 1 ml total volume per culture. IL-2 production in the supernatant was measured by ELISA. MD.45-pLRNL is the mock-transduced MD.45 CTL (MD.45-mock). MD.45-HER/*ζ* or MD.45-mock CTL cultured in the absence of tumour targets produced less than 20 pg ml^−1^ IL-2. Tumour cells cultured alone produced less than 15 pg ml^−1^ IL-2.

b HER-2/neu expression was determined as described in ‘Materials and Methods’.

c Transfected ALC and FM3 cells to express HER-2/neu (ALC-HER, FM3-HER) and their mock transfectants (ALC-mock, FM3-mock).

d Mean values±s.d. from three independently performed experiments.

e Percent inhibition of IL-2 production in the presence of 10 *μ*g ml^−1^ of anti-HER-2/neu mAb (Neu 24.7), added at culture initiation, was calculated as follows: **IL-2 pg/ml w/o mAb−IL-2 pg/ml with mAbIL-2 pg/ml w/o mAb** × 100.

f Primary tumour cells collected from peritoneal effusions during routine aspirations from patients with metastatic breast (Br-1, Br-2) and ovarian (OVA-1, OVA-2) cancer.

, 24 h-incubation of MD.45-HER/*ζ* cells with the SKOV3 and SKBR3 HER-2/neu^+^ tumour cell lines resulted in secretion of relatively high levels of IL-2 (40–100 fold higher compared to background levels (i.e., those achieved with the MD.45-mock cells). That the interaction between MD.45-HER/*ζ* CTL with the tumour cell target was specific for HER-2/neu was best shown using the HER-2/neu transfectants. Thus, recognition of both ALC-HER and FM3-HER by the MD.45-HER/*ζ* cells resulted in 10-fold higher secretion of IL-2 as compared to the IL-2 levels secreted upon recognition of their mock transfectants. Recognition of HER-2/neu^+^ primary tumour cells isolated from patients' ascites led to 15- to 30-fold increased secretion of IL-2 levels as compared to background levels (Table 1). Finally, interaction of MD.45-HER/*ζ* cells with HER-2/neu^−^ K562 and Raji cell lines induced only marginal IL-2 secretion comparable to background levels. The specificity of interaction between MD.45-HER/*ζ* hybridoma CTL and HER-2/neu expressing tumour cell lines or primary tumour cells was also confirmed by the fact that an anti-HER-2/neu MAb when present throughout the 24 h incubation period substantially blocked IL-2 secretion (range of % inhibition: 64–90) (Table 1).

A similar profile for IFN-*γ* secretion was observed in the same cultures. Thus, the SKBR3 and SKOV3 HER-2/neu-overexpressing tumour cell lines, the HER-2/neu transfectants or the primary HER-2/neu^+^ tumour cells stimulated increased secretion of IFN-*γ* by the MD.45-HER/*ζ* transduced CTL hybridoma (Table 2

**Table 2:** Optimal IFN-*γ* secretion of transduced MD.45-HER/*ζ* CTL upon target cell interaction

	**IFN-*γ* production (pg ml^−1^)**		
**Targets**	**MD.45-HER/*ζ***	**MD.45-HER/*ζ*+Neu 24.7**	**MD.45-mock**
ALC-mock	<10	<10	<10
ALC-HER	87±7	23±6 (73)	<10
FM3-mock	<10	<10	<10
FM3-HER	69±9	<10	<10
SKBR3	290±38	45±12 (84)	<10
SKOV3	320±42	72±16 (77)	<10
OVA-1	95±13	29±7 (69)	17±2
OVA-2	165±28	68±6 (59)	<10
Br-1	77±6	23±3 (70)	<10
Br-2	92±7	28±6 (69)	20±6
K562	<10	<10	<10
Raji	<10	<10	<10

See footnotes of Table 1. IFN-*γ* production of either cell type (i.e., transduced MD.45 cells or tumour cell targets) was always <10 pg ml^−1^.

). IFN-*γ* secretion was also greatly inhibited in the presence of the anti-HER-2/neu MAb (% range of inhibition: 69–85) (Table 2).

### Cytotoxic activity of MD.45-HER/*ζ* cells

**Figure 4:**
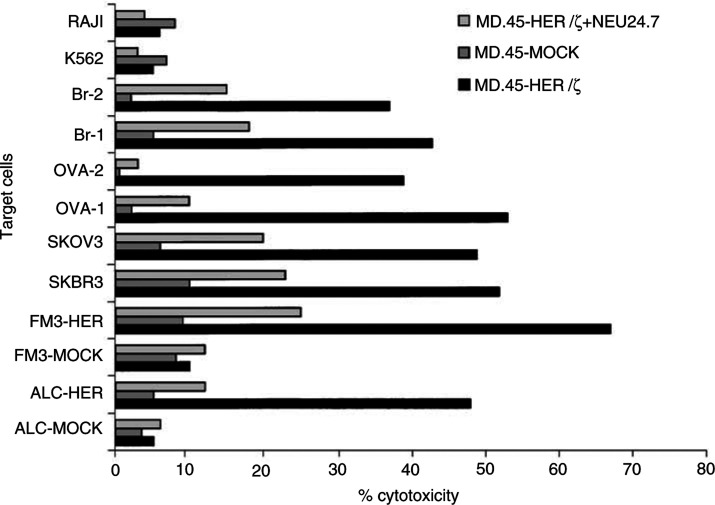
Cytotoxicity of MD.45-HER/*ζ* and MD.45-mock cells against HER-2/neu^+^ (ALC-HER, FM3-HER, SKBR3, SKOV3, Ova-1, Ova-2, Br-1, Br-2) and HER-2neu^−^ (ALC-mock FM3-mock, K562, Raji) targets tested in a 4 h cytotoxicity assay. Blocking of cytolysis mediated by MD.45-HER/*γ* cells was performed with an anti-HER-2/neu MAb (Neu 24.7) at a concentration of 10 *μ*g ml^−1^. The specific ^51^Cr release is depicted at E/T:20. Mean values from triplicate cultures are shown. The s.d. (not shown) never exceeded 10% of the mean values. Results from one representative experiment out of three performed are shown.

The functional expression of the scFv(anti-HER-2/neu)/*ζ* receptor on transduced MD.45 CTL hybridoma was further tested in cytotoxicity experiments against a panel of HER-2/neu^+^ and HER-2/neu^−^ tumour cell lines. As shown in Figure 4

, all tumour targets expressing HER-2/neu were lysed at relatively high levels (range of % cytotoxicity at E/T ratio of 20: 37–67). Lysis of HER-2/neu^−^ tumour targets was always at very low levels and ranged between 5 and 10% (E/T:20). That indeed the specificity of lysis was governed by functional expression of the scFv(anti-HER-2/neu)/*ζ* chimeric gene was further demonstrated (i) by the inhibition of lysis of HER-2/neu^+^ targets following the addition of the anti-HER-2/neu MAb (range of % inhibition: 55–92) and (ii) by the inability of MD.45-mock effectors to lyse HER-2/neu^−^ targets. Finally that the killing mediated by MD.45-HER/*ζ* was non-MHC-restricted can be concluded from the fact that HER-2/neu^+^ targets from unrelated allogeneic donors were as efficiently lysed as the murine ALC-HER cells.

### Treatment of HER-2/neu^+^ tumour cells in SCID mice

**Figure 5:**
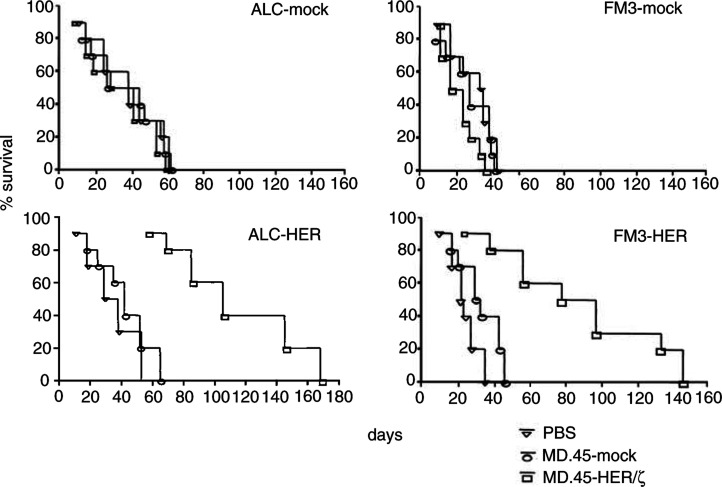
Survival of SCID mice after subcutaneous injection (day 1) with human melanoma FM3 (FM3-HER, FM3-mock) or murine leukaemic ALC (ALC-HER, ALC-mock) cell lines. On the same day (day 1) and for the following 2 days (days 2 and 3) mice were treated with PBS or MD45 cells transduced either with scFv(anti-HER-2/neu)/*ζ* (MD.45-HER/*ζ*) or with their mock tranfectants (MD.45-mock). In all cases, mice treated with MD.45-HER/*ζ* demonstrated a significant increase in survival compared to the other groups (*P*<0.01).

To assess whether MD.45-HER/*ζ* cells had significant *in vivo* activity against HER-2/neu^+^ tumour cells, 1 × 10^6^ murine ALC-HER lymphoma cells or an equal number of human FM3-HER-melanoma cells were inoculated s.c. in mice that had been irradiated (200 rad) 1 day before to suppress endogenous NK activity (Dorshkind *et al*, 1985; Lu *et al*, 1994). On the same day with tumour cell inoculation and for the following 2 days, mice were treated with i.p. injections (one injection per day) of MD.45-HER/*ζ* or MD.45-mock-transduced cells. Mice treated with MD.45-HER/*ζ* cells experienced a significantly prolonged survival as compared to those treated with MD.45-mock cells (for both models >100 days; *P*<0.001) (Figure 5

). The specificity of recognition of ALC-HER or FM3-HER *in vivo* by the MD.45-HER/*ζ* cells was shown by the inability of the later to prevent ALC-mock or FM3-mock growth in the SCID mice (Figure 5).

## DISCUSSION

The HER-2/neu oncoprotein may serve as an excellent target for developing anticancer agents specific for HER-2/neu overexpressing cancer cells. This is based on previously published observations confirming its *in vivo* and *in vitro* role as an immunogenic protein. Thus, CTL responses specific for MHC class I HER-2/neu epitopes have been observed in some patients with cancer and HER-2/neu-specific IgG antibodies have been detected in the sera of patients with HER-2/neu^+^ cancers (Disis *et al*, 1994a,1994b; Yoshino *et al*, 1994;Knutson *et al*, 2001). Furthermore, tumour-reactive CTL- and helper T-cell responses could be induced *in vitro* using various recently identified MHC class I- or class II-binding peptides (Peiper *et al*, 1997; Brossart *et al*, 1998; Kobayashi *et al*, 2000; Sotiriadou *et al*, 2001; Baxevanis *et al*, 2002; Perez *et al*, 2002). Adoptive cellular immunotherapy utilizing patients' T lymphocytes primed *in vitro* to recognise HER-2/neu epitopes represents one major modality for treating patients carring HER-2/neu^+^ tumours. In preclinical models, infusion of HER-2/neu reactive T cells in animals developing HER-2/neu^+^ tumours has led to tumour eradication (Knutson *et al*, 2002). However, adoptive T-cell therapy in patients with HER-2/neu^+^ cancers was relatively unsuccessful due, in part, to the inability to expand tumour antigen-specific T- cells *ex vivo*.

To circumvent the limitations associated with the expansion of HER-2/neu^+^ tumour-specific T cells, we have adopted an approach in which T lymphocytes are grafted with a permanent antibody-dictated specificity (Eshhar *et al*, 2001). To this end, we have provided the *ζ*-chain of TCR with an extracellular recognition domain (scFv) from an anti-HER-2/neu MAb produced by the HB8696 hybridoma. This chimeric construct was stably integrated and expressed by retroviral gene transfer in the MD.45 CTL hybridoma. MD.45 cells transduced to express the scFv(anti-HER-2/neu)/*ζ* could specifically recognise HER-2/neu^+^ breast and ovarian tumour cell lines as well as HER-2/neu^+^ primary tumour cells from patients with metastatic breast and ovarian cancer. Such recognition led to lysis of HER-2/neu^+^ tumour targets and also to secretion of IL-2 and IFN-*γ* by the MD.45-HER effector CTL. The combined induction of cytokine secretion and cytotoxicity by the redirected MD.45 cells is an important issue supporting the use of our chimeric receptor gene for transducing patients' PBL to be used in cellular adoptive immunotherapy. In this way, besides direct tumour cell lysis by transduced CD8^+^ T cells, release of cytokines by transduced CD4^+^ T cells upon specific target cell interaction may also contribute to tumour growth inhibition as well as induction of NK/LAK activities. These lytic activities may result in elimination of tumour cells that lack- or downregulate the relevant (i.e., HER-2/neu) tumour-associated antigen (Pawelec *et al*, 1997).

Chimeric constructs containing a single-chain antibody directed against the human ErbB-2 receptor have been previously demonstrated to redirect T-cell specificity towards mouse epithelial cells transformed to express the human ErbB-2 and human ErbB-2^+^ MDA-MB453 breast cancer cells (Moritz *et al*, 1994; Altenschmidt *et al*, 1997). In these studies, the recognition function was contributed by the scFv domain derived from the MAb FRP5 (Wels *et al*, 1992), specific for the extracellular domain of ErbB-2, whereas the *ζ*-chain of the TCR represented the signalling component. In the present study, we designed a new construct using the scFv from another anti-HER-2/neu Mab produced by the HB8696 hybridoma. Competitive inhibition studies on HER-2/neu^+^ tumours utilizing both FRP5- and HB8696-derived scFvs (one FITC-conjugated and the other nonlabelled) could probably clarify whether these recognise the same or different HER-2/neu epitopes. However, irrespective of the epitope recognised, our MD.45 effectors transduced to express this particular scFv (fused to the TCR *ζ*-chain) were capable of recognising and lysing HER-2/neu^+^ tumour cell lines from different types of cancer as well as primary HER-2/neu^+^ tumour cells from metastatic breast and ovarian cancers. Given the fact that MD.45 cells expressing this construct were also active *in vivo* by inhibiting the growth of HER-2/neu^+^ tumour cells (see below), it becomes evident that our HER/*ζ* construct offers a novel and promising tool for future therapeutic interventions in cancer.

The *in vivo* antitumour activity of MD.45-HER/*ζ* was evaluated in SCID mice/tumour models. Murine ALC or human FM3 tumour cells either transfected to express HER-2/neu or mock-transfected were inoculated into SCID mice. MD.45-HER/*ζ* cells, when implanted simultaneously with the HER-2/neu^+^ tumour cells and for the following 2 days (one injection per day) considerably slowed the growth of these tumour cells. MD.45-HER/*ζ* cells did not exhibit any therapeutic effect when mice were inoculated with ALC or FM3 tumour cells not expressing HER-2/neu (i.e., ALC-mock or FM3-mock cells) indicating the specificity of the *in vivo* antitumour response. This was confirmed by demonstrating the inability of MD.45-mock cells to slow the growth of ALC-HER or FM3-HER tumour cells in SCID mice. Another interesting point which came out from our studies is that the MD.45-HER/*ζ* cells must have the ability to traffic and target tumour cells *in vivo*, since these were given i.p., whereas both the ALC and FM3 tumour cells were given s.c. It is also important to note that the *in vivo* antitumour activity was observed in the absence of exogenously added IL-2 demonstrating that the IL-2 (and most probably also IFN-*γ*) produced by the MD.45-HER/*ζ* cells upon encounter of the tumour cells (as shown in the *in vitro* experiments) was sufficient for supporting their *in vivo* activity. This is an important issue since, clinically, IL-2 administration has been associated with significant toxicity (Rosenberg *et al*, 1994). Finally, although survival was enhanced three-fold, all mice eventually died from their ALC-HER^+^ or FM3-HER^+^ tumours. FACS analysis of these tumour cells showed continued presence of HER-2/neu expression (data not shown). This suggests that antigen downregulation was not the mechanism of escape in this particular model. Another possibility, although not tested herein, could be that the injected MD.45-HER/*ζ* effectors underwent IL-2 dependent, Fas-mediated activation-induced cell death.

Complete tumour eradication may require repeated treatment, combination of i.p. and i.v. therapy, or combinations with other treatment approaches. With respect to this, therapy strategies using a second round of injections with MD.45-HER/*ζ* cells 1 month after tumour cell inoculation or combined treatment with HER-2/neu peptide-specific CTL and MD.45-HER/*ζ* cells are under investigation in our laboratory. Tumour therapy using a variety of chimeric receptors targeting different antigens may also be necessary should antigen downregulation or *in vivo* immunoselection of HER-2/neu negative cells becomes evident.

In summary, the data from this report describe a novel chimeric receptor construct for the gene therapeutic approach to HER-2/neu^+^ cancers. The cells expressing this chimeric construct respond specifically to HER-2/neu^+^ tumours *in vitro* and are also therapeutically effective *in vivo*. Current efforts are focused on enhancing transduction efficiencies and chimeric gene expression in primary T cells, as well as bone marrow stem cells, to maximise the applicability of this technology.
